# Phase I study of the histone deacetylase inhibitor entinostat in combination with 13-*cis* retinoic acid in patients with solid tumours

**DOI:** 10.1038/bjc.2011.527

**Published:** 2011-12-01

**Authors:** R Pili, B Salumbides, M Zhao, S Altiok, D Qian, J Zwiebel, M A Carducci, M A Rudek

**Affiliations:** 1 The Sidney Kimmel Comprehensive Cancer Center at Johns Hopkins, 1650 Orleans Street, CRB1 Room 1M52, Baltimore, MD 21231, USA; 2 NCI-CTEP, Bethesda, MD, USA

**Keywords:** histone deacetylase inhibitor, retinoic acid receptor, solid tumours

## Abstract

**Background:**

Preclinical studies suggest that histone deacetylase (HDAC) inhibitors may restore tumour sensitivity to retinoids. The objective of this study was to determine the safety, tolerability, and the pharmacokinetic (PK)/pharmacodynamic (PD) profiles of the HDAC inhibitor entinostat in combination with 13-*cis* retinoic acid (CRA) in patients with solid tumours.

**Methods:**

Patients with advanced solid tumours were treated with entinostat orally once weekly and with CRA orally twice daily × 3 weeks every 4 weeks. The starting dose for entinostat was 4 mg m^−2^ with a fixed dose of CRA at 1 mg kg^−1^ per day. Entinostat dose was escalated by 1 mg m^−2^ increments. Pharmacokinetic concentrations of entinostat and CRA were determined by LC/MS/MS. Western blot analysis of peripheral blood mononuclear cells and tumour samples were performed to evaluate target inhibition.

**Results:**

A total of 19 patients were enroled. The maximum tolerated dose (MTD) was exceeded at the entinostat 5 mg m^−2^ dose level (G3 hyponatremia, neutropenia, and anaemia). Fatigue (G1 or G2) was a common side effect. Entinostat exhibited substantial variability in clearance (147%) and exposure. CRA trough concentrations were consistent with prior reports. No objective responses were observed, however, prolonged stable disease occurred in patients with prostate, pancreatic, and kidney cancer. Data further showed increased tumour histone acetylation and decreased phosphorylated ERK protein expression.

**Conclusion:**

The combination of entinostat with CRA was reasonably well tolerated. The recommended phase II doses are entinostat 4 mg m^−2^ once weekly and CRA 1 mg kg^−1^ per day. Although no tumour responses were seen, further evaluation of this combination is warranted.

Vitamin A and its active metabolites, including retinoic acid (RA), are essential for growth and cell differentiation of epithelial tissue. Retinoids exert their effects mainly via the nuclear receptors, RA receptor (RAR) and retinoid × receptor, both of which are members of the nuclear receptor super family (Chen *et al*, 1996; Altucci and Gronemeyer, 2001). The human *RARβ* gene is expressed as three isoforms: *β1*, *β2*, and *β4*. The biologically active *RARβ2* isoform is associated with the transcriptional activation of *RARβ2* by RA via binding of RA response elements at its promoter region in a variety of cells. As a tumour suppressor gene, loss of *RARβ* has been associated with cancer progression and hence has been shown to be a potential antineoplastic therapeutic target (Altucci and Gronemeyer, 2001).

Histone acetylation regulates gene transcription (Marks *et al*, 2001). There are two classes of enzymes that can affect the acetylation of histones – histone acetyltransferases and histone deacetylases (HDACs). A number of HDAC inhibitors have been developed that have been shown to inhibit tumour growth *in vitro* and *in vivo* (Ellis *et al*, 2009). For example, vorinostat and romidepsin have been approved for the treatment of cutaneous T-cell lymphomas and have shown to be clinically efficacious (Olsen *et al*, 2007; Piekarz *et al*, 2009). In addition, the synthetic benzamide derivative entinostat (SNDX-275, formerly MS-275) has been found to have selective class I HDAC inhibitory activity and to exert anti-proliferative effects in several tumour models (Saito *et al*, 1999), with a recommended dose established at 4 mg m^−2^ given weekly for 3 weeks every 28 days or at 2–6 mg m^−2^ given once every other week (Gore *et al*, 2008).

Previous clinical trials involving retinoids as single agents have shown no significant clinical activity in patients with epithelial cancer (Altucci and Gronemeyer, 2001). However, combination therapies targeting the *RARβ* gene have generated much interest. There is evidence that the *RARβ2* promoter in epithelial tumours is epigenetically silenced, and that this is reversed by HDAC inhibition, demethylation of DNA at the promoter site, or expression of COUP-TF – an orphan receptor that appears to be required for *RARβ2* promoter response to RA (Lin *et al*, 2000; Bovenzi and Momparler, 2001). Our group and others have reported that chromatin remodelling agents, such as HDAC inhibitors, reverse epigenetic repression of the partially methylated *RARβ2* promoter in epithelial tumours, including prostate, breast, melanoma, and kidney cancer (Sirchia *et al*, 2000; Widschwendter *et al*, 2000; Pili *et al*, 2001; Touma *et al*, 2005; Wang *et al*, 2005; Kato *et al*, 2007). Combination of HDAC inhibitors and retinoids is associated with restoration of *RARβ* in tumours with a partially methylated promoter and greater antitumour activity as compared with single agents (Sirchia *et al*, 2000; Widschwendter *et al*, 2000; Pili *et al*, 2001; Touma *et al*, 2005; Wang *et al*, 2005; Kato *et al*, 2007). Thus, by designing a targeted therapy with *RARβ* agonists and chromatin remodelling therapeutic agents, the preclinical data suggest that it is conceivable to restore retinoid sensitivity in retinoid-resistant tumours with partial *RARβ* promoter methylation. In the presence of HDACs and histone deacetylation, the transcription activating complex (TAC) is unable to bind the promoter of *RARβ* and to induce transcription (Figure 1A). However, in the presence of HDAC inhibitors, TAC binding occurs and transcription is turned on. Taken together, preclinical and clinical data suggest that retinoid-resistant tumours with epigenetic changes at *RARβ2* may benefit from a combined therapy with *RARβ* agonists and chromatin-remodelling drugs such as HDAC inhibitors.

In this study, we tested a targeted transcriptional therapy to enhance/restore retinoid response in patients with metastatic solid tumours by combination of the HDAC inhibitor entinostat with 13-*cis* retinoic acid (CRA). The choice of a selective HDAC inhibitor was primarily based on the availability of entinostat through CTEP. Preclinical studies did not show a difference between class I and class I/I HDAC inhibitors in regards to modulation of RAR*β* re-expression. The objectives of this trial were to determine the dose-limiting toxicities (DLT), maximum tolerated dose (MTD), and pharmacokinetics (PK) of oral entinostat in combination with CRA. We also evaluated the pharmacodynamic (PD) effect of entinostat on target protein expression in peripheral blood mononuclear cells (PBMCs) and in tumour tissue.

## Materials and methods

### Eligibility criteria

Patients with histologically confirmed malignancies without conventional treatment options were eligible. Inclusion criteria included age ⩾18; ECOG performance status ⩽2; life expectancy >3 months; at least 4 weeks elapsed since prior chemotherapy or radiation therapy (6 weeks if the regimen included nitrosoureas or mitomycin C); and adequate haematologic, hepatic, and renal function. This included: absolute neutrophil count ⩾1500 *μ*l^−1^, platelets ⩾100 000 *μ*l^−1^, WBC ⩾3000 cells mm^−3^, haemoglobin >9 g d^−1^, creatinine ⩽1.5 × ULN or measured creatinine clearance of ⩾60 ml min^−1^ per 1.73 m^2^, total bilirubin ⩽1.5 times upper limit of normal, and AST (SGOT)/ALT (SGPT) ⩽2.5 times upper limit of normal. Patients were excluded if they were pregnant or lactating women, had known brain metastases, malabsorption, HIV infection, or serious concurrent medical conditions. Written informed consent, approved by the Institutional Review Board at The Sidney Kimmel Comprehensive Cancer Center was obtained from all patients.

### Dosage and dose escalation scheme

**Figure 1:**
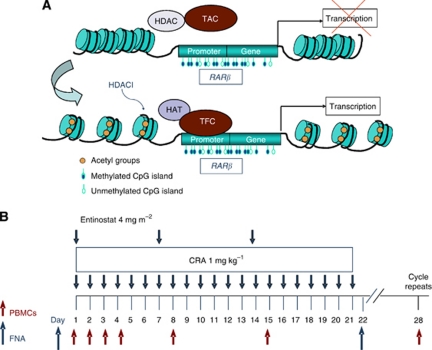
(**A**) Proposed model of epigenetic modulation at *RARβ* gene loci*. RARβ* gene expression is silenced due to histone deacetylation, partial promoter methylation at the CpG islands and associated recruitment of the TAC, making the transcriptional site inaccessible and resistant to retinoid ligands. However, in the presence of HDAC inhibitors (HDACI), *RARβ* is re-expressed, and tumour cell sensitivity to retinoids is restored. (**B**) Treatment schema. Depicted is the schedule of administration of weekly oral entinostat at the starting dose of 4 mg m^−2^ and daily oral CRA 1 mg kg^−1^ for 21 days with 1-week rest. During Cycle 1, FNA was planned for accessible tumours at pre-treatment and day 22.

The dosing schedule was once weekly oral administration of entinostat and daily CRA for 21 days and 7-day recovery period, constituting a 28-day cycle (Figure 1B). A starting dose of 4 mg m^−2^ and a fixed CRA dose at 1 mg kg^−1^ per dose level were planned. If a patient experienced toxicities the next lower level was 2 mg m^−2^. For the dose escalation phase the planned increments of entinostat were by 1 mg m^−2^ at the time. At the time of the original design, the entinostat starting dosage selected for this trial was 2 mg m^−2^. By further review of the existing Phase I safety data on entinostat and the preliminary animal toxicities with this combination the starting dose was then increased to 4 mg m^−2^. Originally six dose levels were included (4–9 mg kg^−1^). Each cohort consisted of three patients according to the standard 3+3 design, where if one patient experiences a DLT, three additional patients are enroled.

Entinostat was supplied as 0.1, 1, and 5-mg tablets by CTEP in conjunction with Schering/AG to the study site. The CRA (Accutane, Roche Laboratories, Nutyley, NJ, USA) was purchased in 10, 20, and 40-mg capsules thanks to an unrestricted grant from Schering/AG. Investigators were trained and authorised to prescribe CRA. A 6–12 patient expansion cohort was pre-planned and was justified to more accurately determine the safe and tolerable dose for further Phase II testing.

### Safety and efficacy measures

At study entry, history, physical examination, laboratory studies (CBC, serum chemistries, liver function tests, and urinalysis), CT scan, chest X-ray, and EKG were performed. Clinical assessments, including a physical examination and adverse event evaluation, were conducted at each follow-up. Adverse events were graded by the NCI Common Toxicity Criteria (Version 3.0). The assessment of toxicities for DLT determination was carried out every week for the first cycle (28 days). Response of measurable lesions was assessed using RECIST criteria (Therasse *et al*, 2000). Tumour markers, when available, were measured but were not included in the tumour response assessment. Laboratory studies for safety were performed on days 1, 8, and 15 of each cycle.

Dose-limiting toxicity was defined as first cycle adverse events ⩾grade 3 non-haematological or ⩾grade 4 haematological toxicities. The MTD was defined as one dose level below the dose at which ⩾ two of six patients experience DLT. In later cycles, dose reduction by one entinostat level (2 mg m^−2^) was applied for the occurrence of either ⩾ grade 3 non-haematological toxicity, grade 4 haematological toxicity, or per the investigator's assessment. Tumour response assessment was performed every two cycles (2 months) by both clinical assessment and imaging studies.

### Drug assay and PK analysis

#### Entinostat

Pharmacokinetic studies were performed during the first 2 cycles of therapy. Serial sampling of venous blood was obtained at the following times during Cycle 1: pre-treatment and at 0.25, 0.5, 1, 1.5, 2, 4, 8, and 10 h after the oral administration of entinostat on day 1. Additional samples were obtained pre-treatment on Cycle 1, days 2, 3, 4, 8, and 15 and Cycle 2, days 1, 15, 16, 17, and 22. Blood samples were collected in heparinised tubes and were processed by centrifugation at 1000 **g** for 10 min. Plasma was stored at −20 °C until analysis. Entinostat concentrations in plasma were measured using a validated LC-MS/MS analytical method over the range of 0.5–100 ng ml^−1^, with a 1 : 10 dilution allowing for quantitation up to 1000 ng ml^−1^ (Zhao *et al*, 2007). Quality control samples were assayed with each analytic run and were within 15% of the nominal concentration. Individual PK parameters were estimated by standard non-compartmental analysis, which was performed using WinNonlin, Version 5.3 (Pharsight Corporation, Mountain View, CA, USA; Gibaldi, 1982).

#### CRA

*Cis* retinoic acid PK studies were performed during the first 2 cycles of therapy. Serial sampling of venous blood was obtained at the following times: pre-treatment on Cycle 1, days 1, 2, 3, 4, 8, and 15 and pre-treatment on Cycle 2, days 1, 15, 16, 17, and 22. Blood samples were collected in heparinised tubes and were processed by centrifugation within 30 min at 1000 **g** at 4 °C for 10 min. Plasma was stored at −20 °C until analysis using a validated LC-MS/MS analytical method. Briefly, 1 ml plasma was extracted with 5 ml of acetonitrile/*n*-Butyl chloride (1 : 4, v/v) containing paclitaxel. *Cis* retinoic acid was separated on a Waters (Milford, MA, USA) X-Terra MS C_18_ (50 × 2.1 mm, 3.5 *μ*m) column with 0.1% formic acid in acetonitrile/10 mM ammonium acetate (80 : 20, v/v) using isocratic flow of 0.2 ml min^−1^ for 5 min. *Cis* retinoic acid was quantitated over the range of 5–2000 ng ml^−1^. Quality control samples were assayed with each analytic run and were within 15% of the nominal concentration. Any sample that was documented to not be a pre-treatment sample (i.e., within 12±4 h after a dose and prior to the next dose) was not utilised in subsequent analysis. *C*_min_ at steady state (*C*_ss,min_) was calculated for each patient by taking the average of the *C*_min_ during each cycle.

### Western blot analyses for protein expression modulation

A total of 10 ml of peripheral blood was collected in a heparinised tube prior to dose 1 of Cycle 1 treatment and 5 ml samples were collected at 2, 8, 24, 48, 72, and 168 h after the first dose of entinostat/CRA treatment. A pre-treatment sample was taken on day 15. During Cycle 2, and subsequent cycles, samples were collected pre-treatment on days 1 and 15. Pharmacodynamic assessment of entinostat on histone acetylation status in PBMCs and tumour tissue was performed by western blot analysis using polyclonal antibodies against human histone H3. Peripheral blood mononuclear cells were collected from patients according to the study calendar schedule. For isolation of PBMCs, 10 ml of blood were collected in CPT tubes (Vacutainer CPT Cell Preparation Tube with NC: 0.45 ml; Ficoll: 1.0 ml; Becton Dickinson, Franklin Lakes, NJ, USA) and plasma was separated by centrifugation. Differential centrifugation of plasma was performed to isolate PBMCs, which were kept frozen at −80 °C until the end of the study schedule for each patient. Fine needle aspiration (FNA) was planned on Cycle 1, day 22 in patients with accessible tumours to assess changes in acetylation and signalling pathway expression. For western blotting, PBMCs and tumour tissue were lysed in M-PER mammalian protein extraction reagent (Pierce, Rockford, IL, USA) with protease inhibitor cocktail (Roche Diagnostics, Indianapolis, IN, USA), followed by brief sonication. Proteins (20 *μ*g per lane) from cell lysates were applied to 4–15% Tris-HCl gels (Bio-Rad, Hercules, CA, USA) and blotted with primary antibodies that were purchased from Upstate (Lake Placid, NY, USA) for anti-acetyl histone H3 and from Sigma (St Louis, MO, USA) for *β*-actin clone A1978, ERK, and phosphorylated ERK.

### Statistical considerations

Descriptive statistics were used to summarise patient characteristics, efficacy, and safety data. Pharmacokinetic parameters were summarised by descriptive statistics using dose-normalised parameters for dose-dependent parameters and actual values for dose-independent parameters. Kruskal–Wallis analysis of variance by ranks or the Wilcoxon rank-sum were used to compare the differences in entinostat PK parameters and CRA *C*_ss,min_ as a function of dose level. Differences between CRA *C*_ss,min_ during each cycle were evaluated statistically by use of a non-parametric, 2-sided Wilcoxon signed rank test for paired observations. Statistical calculations were performed with the software package JMP, Version 4.0.4 (SAS Institute, Cary, NC, USA). The *a priori* level of significance was set at *P*<0.05.

## Results

### General

Over the period of 18 months, 19 patients were enroled on the study. In all, 18 patients received entinostat and were assessable. A total of 17 patients were male, 2 were women. ECOG performance status was 1, except for one patient (ECOG=2). Patients had a minimum of one prior systemic treatment (Table 1). One patient with bladder cancer withdrew before receiving treatment due to disease progression.

### Dose escalation and DLT

Entinostat was given orally without food once weekly (starting at 4 mg m^−2^) and CRA (1 mg kg^−1^) orally twice daily with food for 3 weeks in a 4-week cycle. Dose escalation of entinostat in increments of 1 mg m^−2^ continued until DLT was encountered in 2 or more patients; those who tolerated therapy and did not progress remained on therapy. Dose-limiting toxicities were observed at the second dose level (entinostat 5 mg m^−2^) and included grade 3 hyponatremia (one patient), neutropenia and anaemia (one patient). As the MTD for entinostat was reached at 4 mg m^−2^ (fasting) with 1 mg kg^−1^ CRA, 10 additional patients were treated at this dose level. Three patients required dose reduction to 2 mg m^−2^ secondary to fatigue, neutropenia, and hypophosphatemia in subsequent cycles. No deaths were observed during treatment.

The most commonly reported adverse events were nausea (83% of patients), fatigue (67%), anaemia (61%), dry skin (61%), leucopenia (55%), hypoalbuminemia (55%), and alkaline phosphatase increase (50% Table 2). The majority of adverse events were mild to moderate (grade 1 or 2).

### Pharmacokinetics

*Entinostat* Pharmacokinetics data were obtained for 18 patients, with complete concentration-time profiles available for 17 patients. Summary plasma PK parameters are listed in Table 3. There was no significant difference in the PK parameters between dose levels (*P*>0.05). Median *C*_max_ values reached at 0.42 h. Entinostat concentrations were measurable at 1 week post-treatment in 14 of 17 patients. The mean terminal half-life and apparent oral clearance of entinostat was 113.6±78.2 h and 14.9±22.0 l h^−1^ m^−2^, respectively.

### CRA

Plasma PK studies were completed in 18 patients enroled on the trial and trough values were obtained 78% of the time. Subsequent analysis was performed utilising only the trough values. There was a statistically significant decrease in *C*_ss,min_ between Cycle 1 and Cycle 2 (*P*=0.004), but no difference within a cycle or by dose level (*P*>0.05). The average *C*_ss,min_ during Cycle 1 and Cycle 2 was 194.9±103.5 and 131.8±79.3 ng ml^−1^, respectively.

### Tumour responses

**Figure 2:**
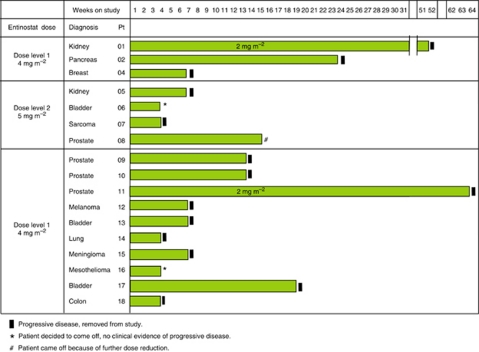
Patient disposition. The graph illustrates the histological tumour types, duration of treatment, and reason for discontinuation in the patients’ cohorts.

The treatment duration for each patient is depicted in Figure 2. Although no complete or partial responses were observed, there were seven cases of stable disease that did not progress after the first two cycles, with durations of 14–63 weeks. For example, one renal cell carcinoma patient (Pt 01) with disease progression following cytokine and anti-angiogenic therapy, initially treated at 4 mg m^−2^ entinostat, had dose reduction to 2 mg m^−2^ after the second cycle. In addition, he achieved sustained stable disease for 12 months with a reduction in the size of lung lesions after 4 months of treatment. Another noteworthy patient (Pt 11) was one with castrate-refractory, chemotherapy-resistant prostate cancer who was initially treated at 4 mg m^−2^ of entinostat, but who also had a dose reduction and was with stable disease for 15 months, and control of his bony pain. Additionally, there was a patient (Pt 02) with unresectable chemotherapy-resistant pancreatic cancer with stable disease for 6 months. These results suggest the effectiveness of this combination in controlling disease progression.

### Pharmacodynamic analyses

**Figure 3:**
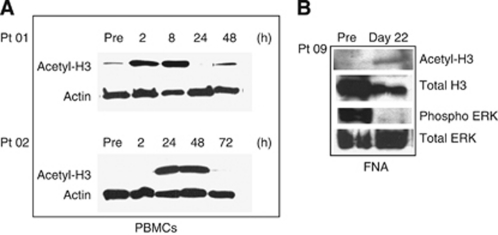
PD analyses. (**A**) Representative western blot analyses for histone H3 acetylation in PBMCs (first two patients enroled in the study). (**B**) Western blot analysis of a liver FNA in a patient with prostate cancer liver metastases.

**Figure 4:**
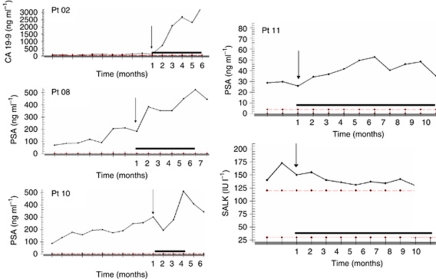
Tumour marker modulation by entinostat and CRA. Representative graphs of PSA and CA 19-9 modulation following treatment with entinostat and CRA in four patients enroled in the study. Concomitant measurement of PSA and serum alkaline phosphatase (SALK) is reported for patient 11. The black bars indicate the time of treatment.

Assayed at pre-treatment and several time points post-treatment, histone H3 hyperacetylation in PBMCs of two representative patients is shown in Figure 3A. Western blot analysis showed transient increased acetylation. Similar general transient histone hyperacetylation was observed in all the patient samples analysed (data not shown). Also shown in Figure 3B is the western blot analysis of a liver FNA of a liver lesion in a patient with metastatic prostate cancer. The analysis showed intra-tumoural induction of acetylated H3 and inhibition of phosphorylated ERK protein levels, indicating targeted inhibition by entinostat and associated downregulation of the MAPK proliferation pathway. Other PD findings are noted in Figure 4. In some patients, we observed elevation of tumour markers such as PSA and CA 19-9 upon starting treatment, with decreases following discontinuation of entinostat and CRA. In one patient (Pt 11), the rise in PSA was associated with a decrease in serum alkaline phosphatase and bony pain.

## Discussion

The efficacy shown by HDAC inhibitors in haematological malignancies has not been confirmed in solid tumours (Byrd *et al*, 2005; Blumenschein *et al*, 2008; Ellis *et al*, 2008; Garcia-Manero *et al*, 2008), and the single-agent development of this class of agents has been disappointing in epithelial tumours. Thus, rational combination strategies are being developed with chemotherapy agents (i.e., paclitaxel, epirubicin), targeted therapies (i.e., epidermal growth factor receptor inhibitors, and vascular endothelial growth factor inhibitors), and radiation treatment (Thurn *et al*, 2011). The purpose of this Phase I study was to define a tolerable and safe dose of entinostat in combination with CRA. This combination strategy was based on previous preclinical models showing re-sensitisation of epithelial tumours to retinoids by HDAC inhibition (Sirchia *et al*, 2000; Widschwendter *et al*, 2000; Pili *et al*, 2001; Touma *et al*, 2005; Wang *et al*, 2005; Kato *et al*, 2007). The CRA dose selected for this trial was 1 mg kg^−1^ per day. This dose was selected based on a number of previous studies showing low toxicity using CRA at this dose level in combination with both cytotoxic and cytostatic therapies. We report, herein, that this combination was relatively well tolerated and, though the observed clinical benefit was limited, the treatment seems to have produced prolonged stable disease in a number of patients with solid tumours.

Entinostat is a selective class I HDAC inhibitor currently in clinical development as either single agent or combination in Hodgkin's lymphoma, breast, non-small cell carcinoma, and myelodysplastic syndrome. A previous Phase I study of entinostat using a 7- or 14-day schedule demonstrated rapid drug absorption with a *T*_max_ reached within 1 h and a large degree of variability in clearance (24–68%) and *C*_max_ (106–285% Gore *et al*, 2008). In this study when administered in combination with CRA, the PK were similar with regards to the *T*_max_, clearance, half-life, and exposure. Previous studies have noted CRA steady-state trough concentrations of 160±19 ng ml^−1^ after administration of a similar dose, suggesting that there is no drug interaction with entinostat (Thurn *et al*, 2011). However, there was some variation between cycles with a significant decrease in trough concentrations noted. This is not surprising since autoinduction of metabolism of another retinoid, all-*trans*-retinoic acid (ATRA), has been noted *in vivo* as a potential source of ATRA resistance (Muindi *et al*, 1992; Agadir *et al*, 1995; Kizaki *et al*, 1996; Conley *et al*, 1997; Accutane, 2000). Overall, a definitive drug interaction is unlikely between entinostat and CRA.

Entinostat/CRA combination treatment in this study did not yield objective responses, however, seven patients did experience stable disease beyond two cycles. One patient, in particular, with heavily pre-treated renal cell carcinoma, had resolution of some lung nodules and had stable disease for 12 months. Another patient with castrate refractory prostate cancer entered the study after progression on docetaxel, and experienced pain relief while on the study and had stable disease by bone scan for 15 months. Of further note, when this patient had to transiently suspend the treatment for a thromboembolic event, he experienced exacerbation of his bone pain until he resumed therapy, suggesting the clinical benefit of entinostat and CRA.

Interestingly, following initiation of treatment with entinostat and CRA, we observed a significantly increased level of tumour markers. The sudden rise of tumour marker CA 19-9 in a patient with unresectable pancreatic cancer was worrisome for a rapid clinical progression. However, the CT scan documented stable disease for 6 months. A similar pattern was observed with PSA levels in our patients with castrate refractory, chemoresistant prostate cancer. An increase in the PSA slope occurred while on treatment, with a rapid deflection following treatment discontinuation. These observations suggest a possible ‘differentiation’ effect of this combination and we hypothesise that tumour marker increases may represent a potential signal for drug exposure, as was shown with another HDAC inhibitor, phenylbutyrate (Gilbert *et al*, 2001). When we tested the activity of achievable plasma concentrations of entinostat on an established human prostate cancer cell line (LAPC4) *in vitro*, there was a 2.5-fold increase in PSA secretion into the culture media (data not shown). However, there was no change in *PSA* gene expression, suggesting that the increase induced by entinostat and CRA was not driven by androgen regulation. This observation poses some challenges as in the clinical development of ‘differentiation’ therapies increasing tumour marker levels may not be a significant sign for disease progression, but rather a potential signal of biological activity. Hence, PSA response should not represent a clinical endpoint in prostate cancer patients treated with chromatin remodelling agents despite some potential bimodal response at higher doses (Rathkopf *et al*, 2010). Accordingly, the use of tumour markers for disease response assessment is evolving and needs to be further validated in prospective studies. Recently, the Prostate Cancer Clinical Trials Working Group recommended that in most clinical trials early changes in PSA should not be acted on without other evidence of disease progression, and treatment should be continued for at least 12 weeks to ensure adequate drug exposure (Scher *et al*, 2008).

David *et al* (2010) recently reported the results of a Phase I trial of intravenous administration of ATRA and the HDAC inhibitor valproic acid (Depakote) in patients with advanced solid tumour malignancies. Unfortunately, the study closed early due to discontinuation of commercial availability of ATRA. Side effects (G2) included skin toxicity and thrombocytopenia. Out of the nine patients treated, the best response was disease stabilisation in one patient with prostate cancer. In this study, no clear overlapping toxicities were encountered with the entinostat and CRA combination and the side effect profile was expected for the two agents. The most common toxicities, as shown in Table 1, were nausea, fatigue, anaemia, dry skin, leukopenia, hypoalbuminemia, and an increase in serum alkaline phosphatase. A MTD was reached and we were not able to dose escalate entinostat to 5 mg m^−2^. Fatigue remains a challenge with chronic use of HDAC inhibitors. In our study, two patients among those who achieved prolonged stable disease had dose reduction to 2 mg m^−2^ after initial cycles, suggesting that clinical benefit may also occur at a dose lower than the recommended 4 mg m^−2^.

Although retinoids are anticancer drugs with clinical activity, their use has been limited by toxicity. Skin toxicities and depression represent the major concerns and the accessibility to retinoids such as accutane is limited. An improved understanding of the structural biology of these nuclear receptors and advances in the chemical synthesis of modified small molecules will lead to the rational design of more selective agents (de Lera *et al*, 2007). For example, selective *RARβ* antagonists may result in decreased skin toxicity by sparing *RARγ*. The combination with chromatin remodelling remains an appealing therapeutic strategy to restore sensitivity when retinoid resistance is due to epigenetic loss of *RARβ2*. We recognise that the assessment of *RARβ*2 expression/methylation in the tumour samples would have strengthened the results of our study. We did perform a liver biopsy in a patient with prostate cancer receiving treatment, but unfortunately we were unable to assess the *RARβ* status. Analysis of *RARβ* in either the primary tumour or the metastatic site should be explored in further testing of this combination.

Though we were unable to assess *RARβ2* expression and modulation and its association with clinical benefit, we did observe signs of intra-tumour target inhibition (induction of histone acetylation) and signal transduction impairment (inhibition of ERK phosphorylation). In general, global histone acetylation in PBMCs has consistently been shown not to be correlated with clinical response, however, intra-tumour histone acetylation and downregulation of ERK is a sign of a direct antitumour effect of this combination.

Combinatorial treatments might lead to synergistic effects on growth control or induction of apoptosis, thereby allowing the use of lower concentrations as well as maintaining efficacy and reducing side effects. Selection of patients who may benefit the most from this approach remains a challenge. On the basis of our findings and others, it is reasonable to speculate that tumours with a re-induction of previously silenced *RARβ2* may be most suitable for this combination strategy. For example, patients with renal and prostate cancer who present with negative *RARβ2* gene expression could be considered for this therapeutic strategy. The answer will likely come from rational preclinical studies and access to tissues from patients receiving differentiation therapies. Some preclinical studies are suggesting potential markers involved in the regulation of growth arrest, apoptosis, signal transduction, metabolism, and immunity (Tavares *et al*, 2008).

In summary, this study reports the results of a Phase I clinical trial to evaluate the safety, PK, and PD of a selective class I HDAC inhibitor, entinostat, in combination with 13-CRA in patients with solid tumours. The combination of a HDAC inhibitor with a retinoid is safe and has the potential to induce clinical benefit in patients with epithelial tumours. Further preclinical and clinical testing will shed light on the mechanism of sensitivity and resistance to retinoids and could help in the selection of patients who may benefit the most from ‘differentiation’ therapies.

## Figures and Tables

**Table 1:** Patient characteristics

Entered/treated	19/18
Male/female	16/2
Median age in years (range)	67 (46–82)
	
*ECOG PS*	
0	7 (39%)
1	5 (28%)
2	1 (5%)
	
*Primary tumour*	
Urothelial carcinoma	5 (28%)
Prostate	4 (22%)
Kidney	2 (11%)
Breast	1 (5%)
Leiomyosarcoma	1 (5%)
Pancreas	1 (5%)
Melanoma	1 (5%)
Lung	1 (5%)
Metastatic meningioma	1 (5%)
Mesothelioma	1 (5%)
Colon	1 (5%)
	
*Prior therapy regimens*	
0–1	4 (22%)
2–3	12 (66%)
4+	3 (16%)
	
Prior radiotherapy, yes/no	14/4

**Table 2:** Adverse events occurring in >20% of patients

**Adverse event**	**All, *n* (%)**	**Grade 1, *n* (%)**	**Grade 2, *n* (%)**	**Grade 3, *n* (%)**	**Grade 4, *n* (%)**
Nausea	15 (83%)	11 (61%)	3 (17%)	1 (6%)	
Fatigue	12 (67%)	2 (11%)	8 (44%)	2 (11%)	
Haemoglobin	11 (61%)	3 (17%)	6 (33%)	1 (6%)	1 (6%)
Dry skin	11 (61%)	7 (39%)	4 (22%)		
Leukocytes	10 (55%)	7 (39%)	2 (11%)	1 (6%)	
Hypoalbuminaemia	10 (55%)	7 (39%)	2 (11%)	1 (6%)	
Alkaline phosphatase	9 (50%)	7 (39%)	1 (6%)	1 (6%)	
Hypophosphataemia	8 (44%)	2 (11%)	3 (17%)	3 (17%)	
Vomiting	8 (44%)	4 (22%)	2 (11%)	2 (11%)	
Platelets	8 (44%)	5 (28%)	2 (11%)	1 (6%)	
Anorexia	7 (39%)	3 (17%)	3 (17%)	1 (6%)	
Neutrophils	6 (33%)		3 (17%)	3 (17%)	
Creatinine	6 (33%)	3 (17%)	3 (17%)		
Hyperglycaemia	5 (28%)	2 (11%)	2 (11%)	1 (6%)	
Hyponatraemia	5 (28%)	4 (22%)		1 (6%)	
Hypocalcaemia	5 (28%)	2 (11%)	3 (17%)		
Headache	4 (22%)	2 (11%)	2 (11%)		
Dry mouth	4 (22%)	4 (22%)			
Taste alteration	4 (22%)	3 (17%)	1 (6%)		

**Table 3:** Pharmacokinetic parameters for entinostat in plasma after Cycle 1, day 1

		**Pharmacokinetic parameters^a^**					
**Dose (mg m** ^−**2**^ **)**	**Number of patients**	***T*_max_ (h)**	***C*_max_ (ng ml** ^−1^ **)**	***V*_z_/*F* (l m** ^−**2**^ **)**	***Cl*_s_/*F* (l h** ^−1^ ** m** ^−**2**^ **)**	***T*_1/2_ (h)**	**AUC_0−∝_ (ng h ml** ^−1^ **)**
4	14	0.50 (0.23–1.50)	114.3±84.0	1368±435^b^	16.8±26.3^b^	129.7±88.5^b^	586.6±401.1^b^
5	4	0.25 (025–1.00)	109.0±66.0	1003±634	10.8±8.0	77.3±31.9	778.4±648.2
Total	18	0.42 (0.23–1.50)		1256±508^c^	14.9±22.0^c^	113.6±78.2^c^	

Abbreviations: AUC_0−∝_=area under the concentration-time curve from time 0 to infinity; *Cl*_s_/*F*, *C*_max_=maximal plasma concentration; *T*_max_=time of the maximal plasma concentration; *T*_1/2_=terminal half-life; *V*_z_*F*=apparent volume of distribution.

a Values are median (range) for *T*_max_ and mean±s.d. for AUC_0−∝_, *C*_max_, *T*_1/2_, and *V*_z_/*F*.

b *n*=9.

c *n*=13.

## References

[bib1] Accutane (Isotretinoin) Capsules Package Insert. Roche Laboratories, Inc.: Nutyley, NJ (2000)

[bib2] Agadir A, Cornic M, Lefebvre P, Gourmel B, Jerome M, Degos L, Fenaux P, Chomienne C (1995) All-trans retinoic acid pharmacokinetics and bioavailability in acute promyelocytic leukemia: intracellular concentrations and biologic response relationship. J Clin Oncol 13: 2517–2523 7595702 10.1200/JCO.1995.13.10.2517

[bib3] Altucci L, Gronemeyer H (2001) The promise of retinoids to fight against cancer. Nat Rev Cancer 1: 181–193 11902573 10.1038/35106036

[bib4] Blumenschein Jr GR, Kies MS, Papadimitrakopoulou VA, Lu C, Kumar AJ, Ricker JL, Chiao JH, Chen C, Frankel SR (2008) Phase II trial of the histone deacetylase inhibitor vorinostat (Zolinza, suberoylanilide hydroxamic acid, SAHA) in patients with recurrent and/or metastatic head and neck cancer. Invest New Drugs 26: 81–87 17960324 10.1007/s10637-007-9075-2

[bib5] Bovenzi V, Momparler RL (2001) Antineoplastic action of 5-aza-2′-deoxycytidine and histone deacetylase inhibitor and their effect on the expression of retinoic acid receptor-*β* and estrogen receptor-*α* genes in breast carcinoma cells. Cancer Chemother Pharmacol 48: 71–76 11488527 10.1007/s002800100294

[bib6] Byrd JC, Marcucci G, Parthun MR, Xiao JJ, Klisovic RB, Moran M, Lin TS, Liu S, Sklenar AR, Davis ME, Lucas DM, Fischer B, Shank R, Tejaswi SL, Binkley P, Wright J, Chan KK, Grever MR (2005) A phase 1 and pharmacodynamic study of depsipeptide (FK228) in chronic lymphocytic leukemia and acute myeloid leukemia. Blood 105: 959–967 15466934 10.1182/blood-2004-05-1693

[bib7] Chen JY, Clifford J, Zusi C, Starrett J, Tortolani D, Ostrowski J, Reczek PR, Chambon P, Gronemeyer H (1996) Two distinct actions of retinoid-receptor ligands. Nature 382: 819–822 8752277 10.1038/382819a0

[bib8] Conley BA, Egorin MJ, Sridhara R, Finley R, Hemady R, Wu S, Tait NS, Van Echo DA (1997) Phase I clinical trial of all-trans-retinoic acid with correlation of its pharmacokinetics and pharmacodynamics. Cancer Chemother Pharmacol 39: 291–299 9025769 10.1007/s002800050575

[bib9] David KA, Mongan NP, Smith C, Gudas LJ, Nanus DM (2010) Phase I trial of ATRA-I V and depakote in patients with advanced solid tumors malignancies. Cancer Biol Ther 9: 678–684 20200483 10.4161/cbt.9.9.11436 PMC3277777

[bib10] de Lera AR, Bourguet W, Altucci L, Gronemeyer H (2007) Design of selective nuclear receptor modulators: RAR and RXR as a case study. Nat Rev Drug Discov 6: 811–820 17906643 10.1038/nrd2398

[bib11] Ellis L, Atadja PW, Johnstone RW (2009) Epigenetics in cancer: targeting chromatin modifications. Mol Cancer Ther 8: 1409–1420 19509247 10.1158/1535-7163.MCT-08-0860

[bib12] Ellis L, Pan Y, Smyth GK, George DJ, McCormack C, Williams-Truax R, Mita M, Beck J, Burris H, Ryan G, Atadja P, Butterfoss D, Dugan M, Culver K, Johnstone RW, Prince HM (2008) Histone deacetylase inhibitor panobinostat induces clinical responses with associated alterations in gene expression profiles in cutaneous T-cell lymphoma. Clin Cancer Res 14: 4500–4510 18628465 10.1158/1078-0432.CCR-07-4262

[bib13] Garcia-Manero G, Assouline S, Cortes J, Estrov Z, Kantarjian H, Yang H, Newsome WM, Miller Jr WH, Rousseau C, Kalita A, Bonfils C, Dubay M, Patterson TA, Li Z, Besterman JM, Reid G, Laille E, Martell RE, Minden M (2008) Phase 1 study of the oral isotype specific histone deacetylase inhibitor MGCD0103 in leukemia. Blood 112: 981–989 18495956 10.1182/blood-2007-10-115873 PMC4081529

[bib14] Gibaldi MPD (1982) Noncompartmental analysis based on statistical moment theory. Marcel Decker (ed). In Pharmacokinetics, pp 409–417. Marcel Dekker: New York

[bib15] Gilbert J, Baker SD, Bowling MK, Grochow L, Figg WD, Zabelina Y, Donehower RC, Carducci MA (2001) A phase I dose escalation and bioavailability study of oral sodium phenylbutyrate in patients with refractory solid tumor malignancies. Clin Cancer Res 7: 2292–2300 11489804

[bib16] Gore L, Rothenberg ML, O’Bryant CL, Schultz MK, Sandler AB, Coffin D, McCoy C, Schott A, Scholz C, Eckhardt SG (2008) A phase I and pharmacokinetic study of the oral histone deacetylase inhibitor, MS-275, in patients with refractory solid tumors and lymphomas. Clin Cancer Res 14: 4517–4525 18579665 10.1158/1078-0432.CCR-07-1461 PMC2813676

[bib17] Kato Y, Salumbides BC, Wang XF, Qian DZ, Williams S, Wei Y, Sanni TB, Atadja P, Pili R (2007) Antitumor effect of combination of retinoic acid with the histone deacetylase inhibitor LAQ824 in malignant melanoma. Mol Cancer Ther 6: 70–81 17237267 10.1158/1535-7163.MCT-06-0125

[bib18] Kizaki M, Ueno H, Yamazoe Y, Shimada M, Takayama N, Muto A, Matsushita H, Nakajima H, Morikawa M, Koeffler HP, Ikeda Y (1996) Mechanisms of retinoid resistance in leukemic cells: possible role of cytochrome P450 and P-glycoprotein. Blood 87: 725–733 8555497

[bib19] Lin B, Chen G, Xiao D, Kolluri SK, Cao X, Su H, Zhang XK (2000) Orphan receptor COUP-TF is required for induction of retinoic acid receptor-*β*, growth inhibition, and apoptosis by retinoic acid in cancer cells. Mol Cell Biol 20: 957–970 10629053 10.1128/mcb.20.3.957-970.2000 PMC85213

[bib20] Marks P, Rifkind RA, Richon VM, Breslow R, Miller T, Kelly WK (2001) Histone deacetylases and cancer: causes and therapies. Nat Rev Cancer 1: 194–202 11902574 10.1038/35106079

[bib21] Muindi JR, Frankel SR, Huselton C, DeGrazia F, Garland WA, Young CW, Warrell Jr RP (1992) Clinical pharmacology of oral all-trans retinoic acid in patients with acute promyelocytic leukemia. Cancer Res 52: 2138–2142 1559217

[bib22] Olsen EA, Kim YH, Kuzel TM, Pacheco TR, Foss FM, Parker S, Frankel SR, Chen C, Ricker JL, Arduino JM, Duvic M (2007) Phase IIb multicenter trial of vorinostat in patients with persistent, progressive, or treatment refractory cutaneousT-cell lymphoma. J Clin Oncol 25: 3109–3115 17577020 10.1200/JCO.2006.10.2434

[bib23] Piekarz RL, Frye R, Turner M, Wright JJ, Allen SL, Kirschbaum MH, Zain J, Prince HM, Leonard JP, Geskin LJ, Reeder C, Joske D, Figg WD, Gardner ER, Steinberg SM, Jaffe ES, Stetler-Stevenson M, Lade S, Fojo AT, Bates SE (2009) Phase II multi-institutional trial of the histone deacetylase inhibitor romidepsin as monotherapy for patients with cutaneous T-cell lymphoma. J Clin Oncol 27: 5410–5417 19826128 10.1200/JCO.2008.21.6150 PMC2773225

[bib24] Pili R, Kruszewski MP, Brandt H, Lantz J, Carducci MA (2001) Combination of phenylbutyrate and 13-cis retinoic acid inhibits prostate tumor growth and angiogenesis. Cancer Res 61: 1477–1485 11245454

[bib25] Rathkopf D, Wong BY, Ross RW, Anand A, Tanaka E, Woo MM, Hu J, Dzik-Jurasz A, Yang W, Scher HI (2010) A phase I study of oral panobinostat alone and in combination with docetaxel in patients with castration-resistant prostate cancer. Cancer Chemother Pharmacol 66: 181–189 20217089 10.1007/s00280-010-1289-x

[bib26] Saito A, Yamashita T, Mariko Y, Nosaka Y, Tsuchiya K, Ando T, Suzuki T, Tsuruo T, Nakanishi O (1999) A synthetic inhibitor of histone deacetylase, MS-27-275, with marked *in vivo* antitumor activity against human tumors. Proc Natl Acad Sci USA 96: 4592–4597 10200307 10.1073/pnas.96.8.4592 PMC16377

[bib27] Scher HI, Halabi S, Tannock I, Morris M, Sternberg CN, Carducci MA, Eisenberger MA, Higano C, Bubley GJ, Dreicer R, Petrylak D, Kantoff P, Basch E, Kelly WK, Figg WD, Small EJ, Beer TM, Wilding G, Martin A, Hussain M (2008) Design and end points of clinical trials for patients with progressive prostate cancer and castrate levels of testosterone: recommendations of the Prostate Cancer Clinical Trials Working Group. J Clin Oncol 26: 1148–1159 18309951 10.1200/JCO.2007.12.4487 PMC4010133

[bib28] Sirchia SM, Ferguson AT, Sironi E, Subramanian S, Orland R, Sukumar S, Sacchi N (2000) Evidence of epigenetic changes affecting the chromatin state of the retinoic acid receptor*β*2 promoter in breast cancer cells. Oncogene 19: 1556–1563 10734315 10.1038/sj.onc.1203456

[bib29] Tavares TS, Nanus DM, Yang X, Gudas LJ (2008) Gene microarray analysis of human renal cell carcinoma: the effects of HDAC inhibition and retinoid treatment. Cancer Biol Ther 7: 1607–1618 18769122 10.4161/cbt.7.10.6584 PMC3060607

[bib30] Therasse P, Arbuck SG, Eisenhauer EA, Wanders J, Kaplan RS, Rubinstein L, Verweij J, Van Glabbeke M, van Oosterom AT, Christian MC, Gwyther SG (2000) New guidelines to evaluate the response to treatment in solid tumors. European Organization for Research and Treatment of Cancer, National Cancer Institute of the United States, National Cancer Institute of Canada. J Natl Cancer Inst 92: 205–216 10655437 10.1093/jnci/92.3.205

[bib31] Thurn KT, Thomas S, Moore A, Munster PN (2011) Rational therapeutic combinations with histone deacetylase inhibitors for the treatment of cancer. Future Oncol 2: 263–283 10.2217/fon.11.2 PMC3127396 21345145

[bib32] Touma SE, Goldberg JS, Moench P, Guo X, Tickoo SK, Gudas LJ, Nanus DM (2005) Retinoic acid and the histone deacetylase inhibitor trichostatin a inhibit the proliferation of human renal cell carcinoma in a xenograft tumor model. Clin Cancer Res 11: 3558–3566 15867260 10.1158/1078-0432.CCR-04-1155

[bib33] Wang XF, Qian DZ, Ren M, Kato Y, Wei Y, Zhang L, Fansler Z, Clark D, Nakanishi O, Pili R (2005) Epigenetic modulation of retinoic acid receptor beta2 by the histone deacetylase inhibitor MS-275 in human renal cell carcinoma. Clin Cancer Res 11: 3535–3542 15867257 10.1158/1078-0432.CCR-04-1092

[bib34] Widschwendter M, Berger J, Hermann M, Muller HM, Amberger A, Zeschnigk M, Widschwendter A, Abendstein B, Zeimet AG, Daxenbichler G, Marth C (2000) Methylation and silencing of the retinoic acid receptor-beta2 gene in breast cancer. J Natl Cancer Inst 92: 826–832 10814678 10.1093/jnci/92.10.826

[bib35] Zhao M, Rudek MA, Mnasakanyan A, Hartke C, Pili R, Baker SD (2007) A liquid chromatography/tandem mass spectrometry assay to quantitate MS-275 in human plasma. J Pharm Biomed Anal 43: 784–787 16971082 10.1016/j.jpba.2006.08.006 PMC1797151

